# Pattern of contributing behaviors and their determinants among people living with HIV in Iran: A 30-year nationwide study

**DOI:** 10.3389/fpubh.2023.1038489

**Published:** 2023-02-24

**Authors:** Zahra Gheibi, Mohammad Fararouei, Sima Afrashteh, Mojtaba Akbari, Parvin Afsar Kazerooni, Mostafa Shokoohi

**Affiliations:** ^1^Department of Epidemiology, Shiraz University of Medical Sciences, Shiraz, Iran; ^2^Clinical Research Development Center, The Persian Gulf Martyrs Hospital, Bushehr University of Medical Sciences, Bushehr, Iran; ^3^Department of Epidemiology and Statistics, Isfahan University of Medical Sciences, Isfahan, Iran; ^4^HIV/AIDS Research Center, Shiraz University of Medical Sciences, Shiraz, Iran; ^5^HIV and Sexually Transmitted Infections Surveillance Research Center, and WHO Collaborating Center for HIV Surveillance Institute for Futures Studies in Health Kerman University of Medical Sciences, Kerman, Iran; ^6^Dalla Lana School of Public Health, University of Toronto, Toronto, ON, Canada

**Keywords:** HIV/AIDS, sexual contacts, injecting drug users, Iran, HIV related behaviors

## Abstract

**Introduction:**

A major shift in the routes of HIV transmission seams to be taking place in Iran. Our study aimed to investigate the 30-year trend of major HIV related behaviors in Iran.

**Methods:**

The national HIV/AIDS registry database (from September 1986 to July 2016 with data on 32,168 people newly diagnosed with HIV) was used to study the 30 years trend and demographic determinants of major HIV related behaviors.

**Results:**

The highest rate of drug injection (DI) among people living with HIV (PLHIV) was reported during 1996 to 1999 (*p*-for trend < 0.001) while the highest rate of sexual activity by minorities or hard to reach groups was during 2004 to 2011 (*p*-for trend < 0.001). Among males, drug injection was directly associated with being single (OR_single/married_ = 1.34), being unemployed (OR_unemployed/employed_ = 1.94) and having lower level of education (OR_<highschool/≥*highschool*_ = 2.21). Regarding females, drug injection was associated with being housewife (OR_housewife/employed_ = 1.35) and lower level of education (OR_<highschool/≥*highschool*_ = 1.85). In females, condomless sexual contact was more common among those younger (OR_20−29/<20_ = 6.15), and married (OR_married/single_ = 7.76). However, among males those being single (OR_married/single_ = 0.82), being more educated (OR_≥*highschool*/<*highschool*_ = 1.24), and being unemployed (OR_unemployed/employed_ = 1.53) reported more sexual activity by minoritised or hard to reach groups.

**Discussion:**

The pattern of major HIV related behaviors among Iranian males and females have been rapidly changing and people living with HIV (PLHIV) are being diagnosed at a younger age. Health education to younger individuals is an essential HIV controlling strategy among Iranian population. Implementation of surveys in hidden and hard-to-reach populations is also recommended.

## Introduction

Acquired immunodeficiency syndrome (AIDS) is caused by human immunodeficiency virus (HIV) *via* weakening the performance of the immune system (1). According to WHO, till 2021, 40.1 million people were living with HIV, of which, almost 38.4 million are alive (2). Noticeably, it is estimated that about 95% of HIV infections occur in developing countries which HIV related behaviors play a critical role on it. A systematic review in sub-Saharan Africa was conducted to define HIV related behaviors and their associated factors among people living with HIV (PLHIV). In this study 76–100% of sexually active adolescents did not reveal their HIV status to their sexual partners because the fear of stigma and discrimination. They also did not use protection during their sexual relation to prevent the transmission of HIV (3). Another study showed that alcohol or cocaine use increase the prevalence of HIV related behaviors (4).

In terms of the geographical distribution of the infections, Asia (including Iran) is the region with the second highest rate of incidence in the world (5). The first individual with HIV infection in Iran was diagnosed in 1987, a hemophilic child who received contaminated blood products. However, during the next decade, the epidemiology of HIV infection in Iran was drastically changed. The high prevalence of HIV infection among Iranian injecting drug users (IDU) shifted the country's HIV prevalence status from low to concentrated, a significant change that raised serious concerns regarding HIV in Iran (6, 7). Later, according to the “Iranian national risk reduction for high-risk groups program”, the prevalence of HIV infection among female sex workers and street children has reached to an alarming level. However, except for a very few areas, our limited knowledge about the changes in the HIV related behavioral factors among Iranian population is hindering our understanding of the epidemiology and prevention of HIV transmission in Iran (8). Also, the prevalence of related behaviors among people living with HIV, especially among the Iranian minoritised groups (e.g., as men who have sex with men, transgender people and sex workers) is unknown due to the limitted access to these important groups. This is important because, although based on the recent reports, a majority (about 67%) of HIV transmissions in Iran has been related to drug use especially drug injection, a shift is taking place in the route of HIV transmission toward sexual routes (9, 10). As a result, underestanding the pattern of major behaviors which are related to the higher risk of HIV transmission and the determinants of these types of behavior is necessary in order to develop effective control measures. Using the national HIV database, this study aimed to investigate the pattern of major HIV related behaviors and their associated factors in Iran over 30 years.

## Materials and methods

The present population based cross-sectional study, used the national database on people diagnosed with HIV from 1986 to 2016 (11). The data is managed by the Iranian Ministry of Health and Medical Education (MOHME) covering all 31 provinces. In Iran, MOHME integrated the HIV/AIDS program into a broad and coherent structure of the national health care system. The routine initial HIV diagnostic tests include ELISA and Western Blot. The confirmed HIV cases are reported to the regional health centers and then to the AIDS coordination department in MOHME. As the result, the national database takes several years to be completed as after collecting data from all provinces the data goes under huge mining and cleaning procedures to make it ready for any particular research use.

After being registered with the system, every HIV-positive individual is to receive standard treatments and gets routine medical follow-up at least twice a year in local HIV centers. All individuals' data is recorded in an unified online registration system under MOHME after being checked and cleaned by the local registry centers (11). For each HIV case, at the time of diagnosis, data on demographic characteristics and HIV associated behaviors is obtained *via* an interview conducted by trained and experienced health staffs in all counties. The collected information includes age, gender (female or male), level of education (elementary, high school, or above), marital status (married, single), occupation (employed or unemployed/housewife), year of HIV diagnosis, history of addiction (yes, no), and major HIV related behaviors. The predefined major behaviors, include history of drug injection (yes, no), out of marriage sexual contact (yes, no), and other conditions (i.e., mother to child transmission, blood transfusion, having sex with the same sex, occupational exposure, and no reported related behavior).

We used the annual number of new cases to define the trend of risky behaviors during the study period. As each individual could report more than one HIV related behavior, we used a logistic regression model for each of the risk factors separately to define their associated factors. To handle missing data, we used multiple imputation *via* applying Chained Equations (MICE) method before running multivariate logistic regression analysis. Analysis was conducted in SPSS, version 22 and STATA, version 14.0 (Stata Corporation, College Station, TX).

### Ethics

All registered individuals routinely provide consent at the time of registration. The study is approved by the ethics committee of Shiraz University of medical sciences (IR.SUMS.REC.1395.S722).

## Results

### Demographic characteristics of the population

In the present study, the data of 32,168 patients who were diagnosed and registered from September 1986 to July 2016 was used. Of the total individuals diagnosed with HIV, 27,130 (84.3%) were male (Table 1). The median (Q_1_, Q_3_) of age was 33 (12, 13) years and age at diagnosis was inversely correlated with the year of diagnoses. In other words, in recent years PLHIV were diagnosed at younger age (*p*-for trend < 0.001). In general, of the registered cases, 48.0% had an education lower than high school and 49.6%were unemployed. In females, more than half of the individuals had an education lower than high school (54.4%) and majority were married (83.1%) at the time of diagnosis (Table 1).

**Table 1:** Descriptive sociodemographic of individuals living with HIV in Iran (*N* = 32,169).

	**Overall *N* (%)**	**Men *N* (%)**	**Women *N* (%)**	*p*-value^*^
**Gender**				–
Male	27,130 (84.3)	–	–	
Female	5,038 (15.7)	–	–	
**Age group (at diagnosis)**				<0.001
<20	1,052 (3.3)	657 (2.4)	395 (7.8)	
20–29	8,815 (27.4)	7,204 (26.6)	1,611 (32.0)	
30–39	11,743 (36.5)	9,926 (36.6)	1,817 (36.1)	
40–49	4,622 (14.4)	3,943 (14.5)	679 (13.5)	
50+	1,500 (4.7)	1,207 (4.4)	293 (5.8)	
Misisng	4,436 (13.8)	4,193 (15.5)	243 (4.8)	
**Marital status (at diagnosis**)				<0.001
Single	10,282 (32.0)	10,866 (40.1)	417 (8.3)	
Married	15,049 (46.8)	9,865 (36.4)	4,185 (83.1)	
Missing	6,837 (21.3)	6,399 (23.6)	436 (8.7)	
**Education (at diagnosis)**				0.037
Below high school	14,432(48.0)	12,688 (46.8)	2,744 (54.4)	
High school or above	4,765 (14.8)	3,604 (13.3)	1,161 (23.0)	
Missing	11,971 (37.2)	10,838 (39.9)	1,133 (22.5)	
**Occupation (at diagnosis)**				<0.001
Unemployed/ housewife	15,966 (49.6)	12,017 (44.3)	3,949 (78.4)	
Employed	10,275 (31.9)	9,576 (35.3)	699 (13.9)	
Missing	5,927 (18.4)	5,537 (20.4)	390 (7.7)	
**Risky sextial behavior**				<0.001
Yes	7,816 (24.3)	6,846 (25.2)	970 (19.25)	
No	23,413 (72.8)	19,416 (71.5)	3,997 (79.3)	
Missing	939 (2.9)	868 (3.2)	71 (1.41)	
**IDU**				<0.001
Yes	21,291 (66.2)	20,919 (77.11)	372 (7.4)	
No	9,938 (30.9)	5,343 (19.7)	4,595 (91.2)	
Missing	939 (2.9)	868 (3.20)	71 (1.4)	
**Addiction history (at diagnosis)**				<0.001
Yes	18,036 (56.1)	17,414 (54.2)	622 (12.3)	
No	4,689 (14.6)	1,496 (5.5)	3,193 (63.4)	
Missing	9,443 (29.4)	8,220 (30.3)	1,223 (24.3)	

* Based on the chi-square test.

Missing values are not included in the chi-square test.

### The frequency and trend of HIV related behaviors

Generally, the prevalence of HIV related behaviors has been changing over the study period (*P*-for trend < 0.001 for all). For example, the highest rate of drug injection was reported during 1996 to 1999 (OR = 4.15) In contrast, condomless sexual contact and unknown reasons for transmission were significantly increasing in recent years (*P*-for trend < 0.001 for both). On the other hand, during the study period, IDU has decreased significantly among males (*P*-for trend < 0.001), whereas, sexual contact and other HIV related behaviors increased (*P*-for trend < 0.001). In females, the highest rate of condomless sexual behaviors was from 2004 to 2011 (*P*-for trend < 0.008) whereas, IDU and no reported reasons for HIV infection are consistently becoming more common during the study period (Table 2).

**Table 2:** Prevalence and trends of HIV risk categories among registered people with HIV in Iran (*N* = 32,169).

	**IDU** ^**a**^		**Sexual** ^**b**^		**Others** ^**c**^		**Unknown** ^**d**^	
**Overall**	***N*** **(%)**	**OR (95% CI)**	***N*** **(%)**	**OR (95% CI)**	***N*** **(%)**	**OR (95% CI)**	***N*** **(%)**	**OR (95% CI)**
Before 1996	80 (31.0)	0.36 (0.27, 0.47)	81 (31.4)	0.41 (0.32, 0.54)	91 (35.3)	2.76 (2.12, 3.360)	6 (2.3)	0.36 (0.16, 0.81)
1996–1999	1,308 (71.4)	4.15 (3.59, 4.80)	349 (19.1)	0.25 (0.22, 0.29)	107 (5.8)	0.36 (0.29, 0.44)	68 (3.7)	0.67 (0.52, 0.87)
2000–2003	3,964 (68.6)	3.13 (2.88, 3.41)	838 (14.5)	0.18 (0.16, 0.19)	691 (12.0)	0.79 (0.71, 0.87)	282 (4.9)	0.89 (0.76, 1.04)
2004–2007	7,534 (62.3)	3.26 (3.04, 3.50)	2,624 (21.7)	0.34 (0.32, 0.36)	1,464 (12.1)	0.90 (0.83, 0.98)	471 (3.9)	0.78 (0.69, 0.89)
2008–2011	5,733 (54.7)	1.94 (1.81, 2.07)	3,408 (32.5)	0.64 (0.60, 0.69)	967 (9.2)	0.67 (0.61, 0.73)	369 (3.5)	0.71 (0.62, 0.82)
2012–2016	4,045 (42.4)	Ref	3,813 (40.0)	Ref	1,219 (12.8)	Ref	454 (4.8)	Ref
*P*-value trend	<0.001	<0.001	<0.001	<0.001	<0.001	<0.001	0.006	<0.001
**Male**								
Before 1996	79 (34.2)	0.17 (0.13, 0.23)	60 (26.0)	0.50 (0.37, 0.67)	87 (37.7)	2.76 (2.09, 3.65)	5 (2.2)	0.38 (0.16, 0.94)
1996–1999	1,303 (73.3)	2.17 (1.84, 2.56)	311 (17.5)	0.35 (0.31, 0.41)	98 (5.5)	0.30 (0.24, 0.37)	66 (3.7)	0.77 (0.58, 1.01)
2000–2003	3,912 (71.3)	1.67 (1.51, 1.85)	651 (11.9)	0.22 (0.20, 0.24)	659 (12.0)	0.71 (0.63, 0.79)	267 (4.9)	1.01 (0.85, 1.20)
2004–2007	7,390 (66.4)	2.27 (2.07, 2.48)	1,960 (17.6)	0.41 (0.38, 0.44)	1,360 (12.2)	0.83 (0.75, 0.91)	422 (3.8)	0.88 (0.75, 1.02)
2008–2011	5,531 (63.5)	2.12 (1.92, 2.34)	2,078 (23.9)	0.65 (0.60, 0.70)	802 (9.2)	0.62 (0.56, 0.69)	299 (3.4)	0.82 (0.69, 0.97)
2012–2016	3,749 (53.0)	Ref	2,106 (29.8)	Ref	933 (13.2)	Ref	280 (4.0)	Ref
*P*-value trend	<0.001	<0.001	<0.001	<0.001	<0.001	<0.001	0.363	0.008
**Female**								
Before 1996	1 (3.7)	0.23 (0.03, 1.73)	21 (77.8)	0.81 (0.34, 1.91)	4 (14.8)	1.10 (0.38, 3.18)	1 (3.7)	0.42 (0.06, 3.14)
1996–1999	5 (9.3)	0.66 (0.26, 1.67)	38 (70.4)	0.68 (0.37, 1.25)	9 (16.7)	1.35 (0.65, 2.79)	2 (3.7)	0.45 (0.11, 1.86)
2000–2003	52 (20.3)	1.51 (1.09, 2.10)	187 (73.0)	0.61 (0.46, 0.81)	2 (0.8)	0.89 (0.60, 1.31)	15 (5.9)	0.68 (0.39, 1,16)
2004–2007	144 (16.2)	1.27 (1.03, 1.58)	664 (74.7)	0.92 (0.76, 1.12)	32 (3.6)	0.91 (0.71, 1.15)	49 (5.5)	0.69 (0.50, 0.96)
2008–2011	202 (11.4)	0.90 (0.75, 1.10)	1,330 (75.3)	1.27 (1.07, 1.49)	165 (9.3)	0.75 (0.61, 0.92)	70 (4.0)	0.52 (0.39, 0.69)
2012–2016	296 (12.0)	Ref	1,707 (69.3)	Ref	286 (11.6)	Ref	174 (7.1)	ref
*P*-value trend	0.093	0.002	<0.001	<0.001	0.517	0.103	0.002	<0.001

a Injection drug use.

b Both heterosexual and same-sex contacts were included.

c Others inclded: blood transfer, mother to child infection, job expouser, and all other blood transmision routs like dentistry services.

d Individuals registered with unknown risky categoreis.

### Determinants of the HIV related behaviors

The results of logistic regression analysis revealed the adjusted and unadjusted associations between the major HIV related behaviors (IDU and sexual contact) and the demographic factors (Tables 3, 4). Generally, IDU was directly associated with being male (OR_male/female_= 16.83, 95% CI: 14.16–19.99), while sexual transmission was more common in females (OR_female/male_= 7.39, 95% CI: 6.51–8.4) (Tables 3, 4). Among men, being single (OR_single/married_= 1.34 95% CI: 1.13–1.58) and being older (OR_30−39/<20_= 17.25, 95% CI: 12.18–24.44) were directly associated with IDU. However, these factors were not associated with IDU among females. In addition, unemployment and lower education were directly associated with IDU in males (OR_unemployment/employed_= 1.94, 95% CI: 1.65–2.27 and OR_<highschool/≥*highschool*_= 2.21, 95% CI: 1.9–2.58), and females (OR_housewife/employed_= 2.21, 95% CI: 1.90–2.58 and OR_<highschool/≥*highschool*_= 1.37, 95% CI: 1.04–1.82), respectively.

**Table 3:** Socio-demographic and background characteristics associated with the acquisition of HIV through injection drug use in the entire sample.

	**Entire sample**		**Only men**		**Only women**	
	**Univariate OR (95% CI)**	**Adjusted OR (95% CI)**	**Univariate OR (95% CI)**	**Adjusted OR (95% CI)**	**Univariate OR (95% CI)**	**Adjusted OR (95% CI)**
Gender (male vs. female)	30.7 (28.1, 33.4)	16.83 (14.16, 19.99)	–	–	–	–
**Age group (ref:**<**20 yrs)**						
20–29 yrs	7.97 (6.90, 9.22)	9.34 (6.79, 12.85)	11.16 (9.39, 13.25)	13.91 (9.87, 19.61)	2.48 (1.64, 3.75)	1.69 (0.94, 3.06)
30–39 yrs	9.46 (8.2, 10.92)	10.62 (7.7, 14.64)	12.89 (10.88, 15.27)	17.25 (12.18, 24.44)	2.29 (1.51, 3.45)	1.64 (0.92, 2.94)
40–49 yrs	8.82 (7.57, 10.27)	8.77 (6.21, 12.37)	10.13 (8.45, 12.14)	12.57 (8.63, 18.29)	2.78 (1.79, 4.31)	1.86 (1.00, 3.45)
50+ yrs	4.16 (3.50, 4.94)	4.28 (2.92, 6.27)	4.16 (3.40, 5.08)	5.74 (3.81, 8.65)	1.55 (0.90, 2.68)	1.42 (0.68, 2.97)
Marital status (single vs. married)	3.15 (2.95, 3.36)	1.34 (1.14, 1.57)	1.28 (1.18, 1.39)	1.34 (1.13, 1.58)	0.93 (0.70, 1.25)	–^*^
Education (< high school vs. ≥high school)	2.12 (2.00, 2.27)	1.99 (1.73, 2.27)	2.7 (2.50, 3.03)	2.21 (1.9, 2.58)	1.85 (1.47, 2.27)	1.37 (1.04, 1.82)
Occupation (unemployed/ housewife vs. employed)	0.56 (0.53, 0.60)	1.75 (1.52, 2.02)	1.27 (1.17, 1.38)	1.94 (1.65, 2.27)	1.35 (1.06, 1.74)	1.23 (0.87, 1.75)
**Calendar period (ref: 2012–2016)**						
Before 1996	0.36 (0.27, 0.47)	0.21 (0.06, 0.68)	0.17 (0.13, 0.23)	0.24 (0.07, 0.77)	0.23 (0.03, 1.73)	1
1996–1999	4.15 (3.59, 4.80)	1.94 (1.14, 3.3)	2.17 (1.84, 2.56)	2.44 (1.36, 4.36)	0.66 (0.26, 1.67)	0.83 (0.14, 4.78)
2000–2003	3.13 (2.88, 3.41)	1.09 (0.86, 1.4)	1.67 (1.51, 1.85)	1.27 (0.97, 1.66)	1.51 (1.09, 2.10)	0.76 (0.41, 1.42)
2004–2007	3.26 (3.04, 3.5)	1.78 (1.5, 2.13)	2.27 (2.07, 2.48)	2.16 (1.75, 2.66)	1.27 (1.03, 1.58)	1.17 (0.83, 1.65)
2008–2011	1.94 (1.81, 2.07)	1.42 (1.22, 1.64)	2.12 (1.92, 2.34)	1.84 (1.53, 2.20)	0.90 (0.75, 1.10)	0.84 (0.64, 1.11)

* Omited in the adjusted model because of the *P*-value > 0.2 in the univariate model.

**Table 4:** Socio-demographic and background characteristics associated with the acquisition of HIV through sexual transmission route in the entire sample.

	**Entire sample**		**Only men**		**Only women**	
	**Univariate OR (95% CI)**	**Adjusted OR (95% CI)**	**Univariate OR (95% CI)**	**Adjusted OR (95% CI)**	**Univariate OR (95% CI)**	**Adjusted OR (95% CI)**
Gender (women vs men)	10.1 (9.44, 10.9)	7.39 (6.51, 8.40)	–	–	–	–
**Age group (ref:**<**20 yrs)**						
20–29	3.16 (2.69, 3.70)	7.91 (6.26, 10.00)	3.04 (2.43, 3.81)	3.69 (2.73, 5)	16.1 (12.4, 20.9)	6.15 (4.17, 9.09)
30–39	2.73 (2.33, 3.20)	6.49 (5.13, 8.2)	2.76 (2.20, 3.45)	3.04 (2.25, 4.12)	14.22 (11.03, 18.34)	4.64 (3.15, 6.84)
40–49	2.36 (2.00, 2.78)	5.38 (4.21, 6.87)	2.38 (1.89, 2.99)	2.59 (1.89, 3.54)	11.71 (8.73, 15.72)	3.1 (2.03, 4.76)
50+	2.39 (1.98, 2.87)	4.06 (3.09, 5.33)	2.21 (1.71, 2.84)	2.14 (1.52, 3.00)	8.25 (5.83, 11.68)	1.71 (1.06, 2.74)
Marital status (married vs. single)	1.76 (1.67, 1.85)	0.96 (0.89, 1.03)	0.92 (0.87, 0.98)	0.82 (0.80, 0.92)	12.5 (11.1, 16.6)	7.76 (5.56, 10.82)
Education (≥high school vs. < high school)	1.48 (1.39, 1.58)	1.15 (1.06, 1.24)	1.45 (1.35, 1.57)	1.24 (1.14, 1.34)	0.99 (0.82, 1.18)	–^*^
Occupation (unemployed/housewife vs. employed)	1.12 (1.07, 1.18)	1.45 (1.35, 1.55)	1.81 (1.72, 1.92)	1.53 (1.43, 1.65)	1.28 (1.03, 1.6)	1.02 (0.77, 1.35)
**Calendar period (ref: 2012–2016)**						
Before 1995	0.61 (0.45, 0.81)	0.86 (0.39, 1.89)	0.71 (0.52, 0.98)	0.79 (0.35, 1.77)	0.84 (0.30, 2.40)	1.62 (0.08, 33.89)
1996–1999	0.43 (0.33, 0.56)	0.6 (0.48, 0.75)	0.44 (0.32, 0.60)	0.57 (0.46, 0.71)	0.76 (0.31, 1.86)	0.4 (0.14, 1.17)
2000–2003	0.82 (0.63, 1.00)	0.43 (0.38, 0.49)	0.82 (0.61, 1.11)	0.39 (0.34, 0.44)	1.14 (0.48, 2.72)	1.02 (0.61, 1.7)
2004–2007	1.56 (1.19, 2.04)	0.65 (0.59, 0.71)	1.31 (0.97, 1.77)	0.6 (0.54, 0.66)	1.57 (0.66, 3.72)	0.89 (0.66, 1.2)
2008–2011	2.42 (1.85, 3.10)	0.81 (0.74, 0.88)	2.01 (1.49, 2.72)	0.74 (0.67, 0.81)	1.24 (0.52, 2.93)	1.13 (0.90, 1.43)

* Omited in the adjusted model because of the *P*-value > 0.2 in the univariate model.

Condomless sexual contact was associated with younger age (OR_20−29/<20_= 7.91, 95% CI: 6.26–10.00) in both genders whereas, marriage was directly associated with risky sexual contact in females (OR_married/single_= 7.76, 95% CI: 5.56–10.82). In males, the association was inverse (OR_married/single_= 0.82, 95% CI: 0.80–0.92). Unlike for male individuals with HIV, higher education was directly associated with risky sexual contact in females (OR_≤*highschool*/<*highschool*_= 1.24, 95% CI: 1.14–1.34). Also, unemployment in males was directly associated with risky sexual behaviors (OR_unemployment/employment_= 1.53, 95% CI: 1.43–1.65).

## Discussion

In this study, we investigated the pattern of major HIV related behaviors in all registered PLHIV in Iran from 1986 to 2016. According to the results, the most common route of HIV transmission in Iran is drug injection in males and sexual contact in females. However, the above picture is rapidly changing as drug injection is replaced by sexual contact in males and other routs (especially unknown reason) in females. The downward trend of IDU is possibly due to the introduction of synthetic drugs that are becoming more popular among younger individuals, making them less exposed to IDU and shared syringe. However, a higher rate of condomless sexual contact is reported among the Iranian younger population (14, 15). Among the younger Iranian population, factors such as the rising age of marriage, unemployment, and other socioeconomic changes may provide the ground for the significant change in sexual behaviors. It is also suggested that social media, internet, and satellite programs are potential factors affecting the Iranian culture (16). The contradiction between the social values of the traditional societies and the modernized views of the younger population in the Iranian society may rise the above HIV related behavior (17). Moreover, the stigma, fear of ostracism and discrimination may prevent young individuals from asking help and receiving adequate education and protection (17–19). Recent studies indicated that premarital and extramarital sexual relationship among Iranian youth have increased considerably (20, 21). A study in the Middle East and North Africa reported HIV epidemics that appeared among MSMs in few countries (22). In Iran, the epidemic of HIV infection started with blood transfusion but the route of transmission was rapidly evolved to transmission *via* IDU and, in recent years, sexual contacts. In fact, sexual routes are becoming the main route of HIV transmission in Iran. As it was mentioned before, the distribution of gender in PLHIV and the trend of risky behaviors had fluctuations, in Iranian population. For example, before 1995 the primary and main potential reason of HIV transmission in Iran was blood transfusion which was significantly controlled *via* applying safe blood transfusion policies. After 1995, because of both attempts of HIV case finding and the increasing number of the people living with HIV due to IDU, the number of HIV infections raised rapidly among men, with a peak in 2006. Iran is located at the main narcotic transit route in the region and IDU has raised to such a level that it has become the strongest contributing factor of HIV epidemic in the country. The observed later downward trend of HIV infection due to IDU is mainly because of the implementation of IDU-related harm reduction programs e.g., methadone maintenance treatment, needle and syringe distribution, and public health education (19). There is however another possibility that at least a part of the change in the pattern is due to the introduction of synthetic drugs that are easily applicable without need for injection (7, 23, 24). We revealed that although injecting drug use is a common route of HIV transmission among men in Iran, this type of HIV related behavior is quite uncommon among women. As suggested in the present study, women are more vulnerable to condomless sexual contact than men. This vulnerability in women is mainly because they are more involved in (condomless) sex with their infected partners (25). Women are also disproportionately more exposed to HIV due to limited access to proper education on HIV, gender inequality, economic dependency, and gender discrimination (26). Because of these reasons, HIV infection among women is alarmingly raising in Iran.

The results of our study showed that at the time of diagnosis, IDU among men was more common when they were younger. This finding was in line with the literature about the role of age in becoming HIV infected among injecting drug users. For example, a study in Taiwan showed that being younger than 45 years of age is associated with a higher risk of HIV infection. The study concluded that younger people do not have adequate knowledge about HIV infection and common routes of its transmission. As a result, young IDUs should be in the center of HIV prevention and education programs (27). In contrary, being of older age was associated with an increased risk of becoming HIV infected in injecting drug users in another study (12). In the present study, younger age and marital status was associated with condomless sexual relations. We revealed that marriage is a risk factor for females, where as it was protective for males. In this regard, a study on American adults showed that the likelihood of having only one sexual partner decreases with age. Also, the availability of delaying drugs may lead to more prolonged intercourses in older men. Therefore, more people would live with HIV in middle age (28). Negin et al. suggested that some older people seek new sexual relationships that may put them at greater risk for HIV infection (29).

In our study, lower education was associated with drug injection in men and women. A study in China reported that people with higher education who use drugs are more likely to be aware of the risk factors for HIV/AIDS and have more access to harm reduction methods. They were also less likely to accept shared syringes (30). In another study, Hasnain et al. showed that a lower level of education increases the risk of HIV transmission in IDUs (31). However, in our study, people with higher levels of education were more likely to have condomless sexual contacts. This result contradicts those from other studies. For example, in a study on men who have sex with men (MSM), those with a university degree or higher education, were at lower risk of contracting HIV (32).

We showed that unemployment is more common among both men who use drugs and men with condomless out of marriage sexual contacts. However, the association was not significant in women. Likewise, Swe and Rashid showed that unemployment increases the risk of HIV and greater risk of using shared syringes in injecting drug users (33). The authors believe that, financial pressure resulting from unemployment can put a person at higher risk of HIV infection (34). In other words, low income is a predictor of HIV infection because being in disadvantaged social groups can indirectly encourage high-risk behaviors such as sharing needles or condomless sex (35). According to the previous studies on injecting drug users and men with condomless sexual contact, being single is associated with an increased risk of HIV infection (27, 36, 37). It is also reported that, losing partner may make a person to choose relationships that increase the risk of contracting HIV (36).

In summary, low socioeconomic status, low social cohesion, and instability in sexual relationships may justify the increase risk of HIV transmission among single people. Therefore, delivering HIV preventing services including health education and harm reduction measures to single people must be considered seriously (13). Our results revealed important changes in the potential transmission routes of HIV in Iran. Accordingly, the prevention programs in Iran should be revised to effectively reduce the risks of sexual practices.

## Limitations

Because of under-reporting, under-ascertainment, and difficulties in reaching high-risk groups, surveillance of infectious diseases (including HIV) is a big challenge in many countries including Iran (38). As a result, the Iranian HIV database suffers from low HIV case detection rate. Because of social stigma and family concerns, sexual practices or other contributing behaviors which have traditionally been taboo/stigmatized/legislated against in Iranian society, sex workers, MSM, and people who use drugs may refuse to report their HIV contributing behaviors. Another challenge in the present research was the considerable number of missing data in several variables. For the multivariate analysis, we implemented the MCMC method to reduce the effect of the missing data on the results.

## Conclusion

The HIV routes of transmission are changing in Iran. This is mainly the result of the dramatic changes in the lifestyle of younger individuals and change in the major related behaviors, from reduction in unsafe injections to raise in more open sexual relationships. Therefore, the national HIV preventative policy needs to be continuously revised in Iran. Providing health education to people with the related behaviors and young individuals to reduce the risk of HIV transmission and case detection in groups with particular sexual preferences are essential strategies (39). Implementation of several integrated socio-behavioral surveys with valid sampling methods in hidden and hard-to-reach populations is also recommended. Our results can help health policymakers to design and implement aimed and prioritized interventions to reduce HIV transmission in the minoritized groups.

## Data availability statement

The data analyzed in this study is subject to the following licenses/restrictions: The data have a unique code for each cases to protect their privacy. Requests to access these datasets should be directed to fararooei@gmail.com.

## Ethics statement

The study was approved by the Ethics Committee of Shiraz University of Medical Sciences (IR.SUMS.REC.1395.S722). The patients/participants provided their written informed consent to participate in this study.

## Author contributions

ZG was responsible for preparation of the manuscript, measurement methods, interpretation of results, data collection, and data analysis. MF was responsible for coordination and management and manuscripts preparation. SA was responsible for data collection and interpretation of results. MA and PA were responsible for preparation of the manuscript. MS was responsible for measurement methods data analysis. All authors have read and approved the manuscript.
